# Performance of volume and diameter thresholds in malignancy prediction of solid nodules in lung cancer screening

**DOI:** 10.1136/thorax-2024-222086

**Published:** 2025-06-02

**Authors:** Andrew W Creamer, Carolyn Horst, Ruth Prendecki, Priyam Verghese, Amyn Bhamani, Helen Hall, Sophie Tisi, Jennifer L Dickson, Chuen R Khaw, John McCabe, Tanita Limani, Kylie Gyertson, Anne-Marie Hacker, Jonathan Teague, Laura Farrelly, Shrinkhala Dawadi, Neal Navani, Allan Hackshaw, Anand Devaraj, Arjun Nair, The SUMMIT Consortium, Sam M Janes, Sam M Janes

**Affiliations:** 1 Lungs for Living Research Centre, UCL Respiratory, University College London, London, UK; 2 University College London Hospitals NHS Foundation Trust, London, UK; 3 Cancer Research UK and UCL Cancer Trials Centre, University College London, London, UK; 4 Royal Brompton Hospital, London, UK; 5 National Heart and Lung Institute, Imperial College, London, UK

**Keywords:** Lung Cancer, Imaging/CT MRI etc

## Abstract

**Background:**

Prospective validation and comparison of the performance of nodule management protocols is limited. The aim of this study was to examine the performance of size and risk thresholds for assessing malignancy in solid nodules at baseline low-dose CT (LDCT) in a lung cancer screening (LCS) programme.

**Methods:**

This was an observational study using data from the SUMMIT Study, a prospective longitudinal study investigating LDCT for LCS. Participants were 55–77 years old and met either the United States Preventative Services Task Force (2013) criteria or had a PLCO_m2012_ risk of ≥1.3%. LDCTs were reported using computer-aided detection software (Veolity, MeVIS) with semiautomated volumetry. Cancer outcomes were reported for solid nodules reported at baseline CT, with participants represented by the single largest solid nodule where more than one was present. Malignancy risk was stratified by long-axis diameter and volume using predefined size thresholds taken from British Thoracic Society and European Position statement guidelines: a 5/6 mm long axis diameter or 80/100 mm^3^ volume ‘rule out’ thresholds for low-risk nodules and ≥300 mm^3^ or ≥8 mm diameter with or without Brock score ≥10% ‘Rule in’ thresholds for high-risk nodules.

Pearson’s χ^2^ test was used to calculate statistical significance for nominal variables, McNemar’s test for comparison of sensitivity/specificity and DeLong’ test for comparison of areas under the receiver operating characteristic curve (AUROC). Optimal thresholds were determined with Youden’s J statistic. Net benefit calculations were undertaken to compare the existing thresholds with 95% CIs calculated by bootstrap sampling.

**Results:**

11 355 participants were included. Crude risk of malignancy in solid nodules at baseline LDCT was 3.8% (228/5929). Risk of malignancy in solid nodules <6 mm long-axis diameter or <100 mm^3^ volume was equivalent to that in participants with no nodules at baseline LDCT (0.88% and 0.84% vs 0.77%, p=0.4600 and p=0.7932, respectively). A <80 mm^3^ volume and <5 mm diameter ‘rule out’ threshold achieved sensitivity 86.8% and 93.4%, specificity 65.4% and 24.64%, and negative predictive value (NPV) 99.2% and 98.9%, respectively. The <80 mm^3^ volume threshold encompassed 63.3% of participants with a baseline solid nodule compared with 24.0% by the <5 mm diameter threshold.

For nodules ≥8 mm diameter, the addition of a risk score (Brock ≥10%) was associated with a significant net benefit when compared with using size threshold alone by net effect analysis (31.24; 95% CI 26.19 to 35.89).

**Conclusions:**

Solid nodules <100 mm^3^ or <6 mm diameter are not associated with increased risk of lung cancer compared with participants with no nodules at baseline LDCT. Volumetric rule-out thresholds achieve equivalent NPV to long-axis diameter thresholds while encompassing significantly more participants, reducing the number of interval scans required.

**WHAT IS ALREADY KNOWN ON THIS TOPIC**

- Several approaches to risk stratification of solid pulmonary nodules using size or multivariable models have been developed and validated.
- These have been encompassed in nodule management guidelines determining when nodules should undergo surveillance, further assessment with positron emission tomography-CT (PET-CT), or be safely disregarded.
- However, there has been limited prospective assessment of the performance of such guidelines, nor comparative analysis where guidelines differ.

**WHAT THIS STUDY ADDS**

- We present data on the performance of the British Thoracic Society Nodule management guidelines for solid nodules reported at baseline CT; an approach which prioritises volumetric assessment of nodule size and incorporates a risk-prediction model (Brock) when determining referral for PET-CT.
- We compare the performance of this approach to different size thresholds, long-axis diameter compared with volume for size assessment, and the inclusion of a risk prediction model (Brock) in addition to size alone.

**HOW THIS STUDY MIGHT AFFECT RESEARCH, POLICY OR PRACTICE**

- The data presented serve as a benchmark for the performance achievable with a volumetric-based approach to nodule risk stratification and offers insights into the net benefits (trade-off between sensitivity and specificity) for adopting alternative thresholds for nodules undergoing surveillance or referral for PET-CT.

## Background

 Effective lung cancer screening (LCS) with low-dose CT (LDCT) requires the ability to discriminate between pulmonary nodules which are sufficiently suspicious for malignancy to warrant referral for definitive investigation; intermediate risk requiring further surveillance; or of sufficiently low risk to disregard. Several models have been developed and validated for predicting malignancy of a nodule seen on a single or baseline LDCT,^1^,^3^ only some of which have been developed from LCS data.^1 4^ Size is the dominant factor in these models and thus the key discriminator in guiding management at baseline LDCT in LCS programmes. Guidelines have been developed for both screen-detected^5^ and incidentally detected^6 7^ nodules which propose size thresholds below which no further surveillance is required, and also size and risk thresholds above which further investigation (eg, with positron emission tomography CT, PET-CT) should be performed.

There remains discordance between guidelines for the preferred measurement method, size thresholds and the point at which predicted malignancy risk is incorporated into management decisions. The British Thoracic Society (BTS) nodule management guidelines (2015)^6^ using evidence largely from LCS data recommend volumetry as the primary sizing method, with a <80 mm^3^ ‘rule out’ threshold (<5 mm maximum diameter where reliable volumetric analysis cannot be obtained) for solid nodules at baseline LDCT, and is used by the targeted lung health check (TLHC) programme in England.^8^ Lung-RADS (reporting and data systems) (V.2022),^9^ used in screening programmes in the USA, advocates mean diameter as the primary measure of size, and recommends a <6 mm diameter threshold for baseline solid nodules, while the European Union position statement on LCS (EUPS)^10^ recommends a <100 mm^3^ volumetric rule-out threshold. For referral for PET-CT, the BTS guideline recommends assessing malignancy risk with a specific risk calculator (the PanCan model) at a size threshold of 300 mm^3^ or 8 mm in maximum diameter, while Lung-RADS suggests considering PET-CT for any solid nodule >8 mm mean diameter without specific risk assessment mentioned for guiding subsequent investigation until nodules are >15 mm diameter.

To our knowledge, there has been no prospective validation of the discriminatory performance of these thresholds in a large (>10 000 participants) screening cohort. With the recent approval of a national LCS programme by the National Screening Committee in the UK,^11^ assessment of these thresholds is urgently required.

The aim of this analysis was to use data from the SUMMIT Study to (1) Report the performance of the BTS nodule management guidelines in ruling out benign and identifying malignant solid nodules at baseline LDCT; (2) Compare the performance of a nodule management protocol including volumetric (where possible) thresholds to that achieved by single long-axis diameter thresholds; and (3) Compare the performance of the ‘rule out’ volume thresholds used by BTS and EUPS, and of a mathematically determined ‘optimal’ Brock threshold against the current 10% risk (advocated by BTS) for identifying nodules requiring further investigation. This analysis was limited to solid nodules to reflect the differences in measurement methods and diagnostic approach recommended between solid and subsolid nodules in all major guidelines.^6 7 9^

## Methods

### Study design and participants

This analysis is an observational study using data from the SUMMIT Study. The SUMMIT Study is a prospective, longitudinal cohort study which aims to assess the implementation of LDCT screening and validate a multicancer early detection blood test (ClinicalTrials.gov NCT03934866). Invitations and uptake to screening have been described previously.^12^ Participants were 55–77 years old at the time of invitation and met either the United States Preventative Services Task Force 2013 criteria^13^ or had a prostate-lung-colorectal-ovarian risk model (PLCO_m2012_^14^) of ≥1.3%. Eligible participants attended three annual lung health checks (Y0, Y1 and Y2). LDCT screening was performed in all participants at the Y0 and Y2 visits, with participants randomised to scan or no scan at the Y1 visit in a 1:1 ratio (unless the Y0 scan identified a finding which mandated further surveillance at the Y1 timepoint).

Written consent was obtained from the participants. Results from the multicancer early detection blood test were not available to clinicians managing the screening programme and are not considered in this analysis.

### Study procedures

Study scans were performed on GE Revolution multislice scanners, with scans performed at maximal inspiration in one continuous craniocaudal acquisition without intravenous contrast. Thin collimation (0.625 slice thickness) volumetric CT images were reconstructed using a soft tissue algorithm and 50% ASiR-V (adaptive statistical iterative reconstruction). Images were analysed with computer aided detection (CADe) software for semiautomated volume measurements (Veolity V.1.4, MeVIS Medical Solutions, Germany). All images were subsequently reviewed by an experienced thoracic radiologist, who accepted or rejected CADe-identified nodules, assessed reliability of volumetric segmentation and reviewed for clinically relevant nodules not detected by CADe.

Diameter is given as a single long axis and volume as the CADe-derived value (where reliable segmentation could be achieved). Long axis was used for measuring diameter in accordance with BTS guidelines,^6^ rather than the average diameter recommended by Lung-RADS.^9^ Diameter was CADe derived (by longest axis measurable from contoured segmentation), unless reliable nodule segmentation could not be performed in which case manual measurement was recorded. Radiologists also recorded the presence of additional metrics for calculating a Brock Score (emphysema, lobe location and spiculation).

The SUMMIT nodule management protocol is based on British Thoracic Society guidelines.^6^ A volumetric approach was used for solid nodules with diameter only used where reliable segmentation could not be achieved.^15^ Solid nodules at baseline scan of ≥300 mm^3^ (or ≥8 mm diameter) with Brock Score ≥10% were referred for definitive investigation. Solid nodules of ≥80, <300 mm^3^ on baseline scan underwent interval scan at 3 months. Nodules stable at a 3-month interval scan underwent further scans at year 1 and year 2. Nodules demonstrating growth with volume ≥200 mm^3^ were referred to a local multi-disciplinary team (MDT) for definitive assessment.

### Outcomes

The aim of this analysis was to determine the sensitivity, specificity, positive predictive value (PPV) and negative predictive value (NPV) of volumetric and diameter size thresholds in predicting malignancy in solid nodules at baseline LDCT. The primary endpoint of this analysis was the probability of a screen-detected solid nodule being diagnosed as malignant as defined by either outcome from treating hospital following referral from the study or registration of a lung cancer diagnosis on the National Cancer Registration and Analysis Service (NCRAS) data set. While the large majority of malignant nodules represented lung cancer, we also included those subsequently found to represent malignancy other than primary lung cancer (eg, metastases from non-lung primary, pulmonary lymphoma). Cancer outcomes were linked to individual screen-detected nodules by anatomical location at resection. In cases of multiple nodules in the resected lobe/segment, additional investigations (CT-guided biopsy, contrast-enhanced CT and PET/CT images) were reviewed alongside screening images to determine the malignant nodule. In cases of synchronous lung cancers or satellite lesions, only one nodule/cancer was used in the analysis; this allowed results to be presented as a 1:1:1 ratio of participants to nodules to cancer diagnoses. Cancers arising from subsolid nodules or consolidation and any nodule first identified at subsequent screening rounds were not considered in this analysis.

For cancers diagnosed following referral from SUMMIT, malignancy was confirmed by histology or clinicoradiologically by MDT consensus where invasive procedures were declined or felt inappropriate.

Nodule benignity was defined as subsequent resolution, radiological stability over 2 years or if malignancy was excluded following investigation after referral to an MDT. For participants who did not complete the Y2 visit, benignity was assumed by an absence of lung cancer diagnosis for that participant on national cancer registries at 3 years after baseline scan.

Nodules consistent with typical intrapulmonary lymph nodes (solid nodules with juxtapleural location; smooth margins; and oval, lentiform or triangular shape) did not undergo dedicated follow-up,^16^ but were included in this analysis as a non-calcified solid nodule. Radiologists were instructed to report all nodules identified by CADe (quoted detection limit of 4mm long axis diameter^17^), but not to report every micronodule present below the threshold for CADe detection. Nodules which were not reported at baseline LDCT but seen to be present when reviewed retrospectively (when reporting a subsequent study scan), termed ‘retronodules’, were included when they proved malignant (as participants with benign solid nodules were stratified by size of the largest nodule, benign retronodules were not included as we could assume they would be smaller than the reported nodule and therefore wouldn’t alter the stratification of the participant). Nodules were analysed according to dimensions measured at baseline LDCT (ie, for retronodules we used the retrospectively measured dimensions on the baseline LDCT). Participants with baseline LDCT reported as ‘No nodules’ were analysed as ‘cancer risk with no nodules’.

### Statistical analysis

Data are presented at a per-participant level. Participants with more than one solid nodule at baseline CT were represented by the largest (if all benign), or that which proved malignant. Sensitivity, specificity, PPV and NPV were calculated conventionally at the predetermined thresholds specified by various guidelines.^6 9 10^ Risk was expressed compared with participants with ‘no nodules’ reported at baseline CT. Statistical significance for nominal variables was calculated with Pearson’s χ^2^ test, with McNemar’s test used for comparison of sensitivity/specificity. Statistical significance was defined as p<0.05. Areas under the receiver operating characteristic curve (AUROC) were compared for statistically significant difference with DeLong’s test and Youden’s J statistic used to determine optimal thresholds.

To quantify the net effect of different thresholds (ie, trade-offs between sensitivity and specificity), a net effect analysis was performed using the formula:^18^

Net benefit = ΔSensitivity + (ΔSpecificity *(1/W) *(1−p)/p)

Where p is the prevalence and W is a weighting factor for change in specificity, with a higher W implying a lesser effect of a given gain in specificity (prioritising sensitivity).^19^ A positive outcome where the 95% CI does not cross zero implies a statistically significant benefit for the comparison threshold. We assigned a weighting factor of 50 for assessment of the ‘rule out’ threshold (ie, in 50 participants undergoing nodule follow-up CT, 1 cancer was diagnosed), and five for the ‘rule in’ threshold (ie, in 5 participants referred for PET-CT, 1 cancer was diagnosed) on the basis of a screening programme with a 2% cancer prevalence which used a 3-month interval scan for indeterminate nodules (with subsequent growth identifying malignancy^20^). 95% CIs for the net benefit estimates were calculated by bootstrap sampling of 1000 generated samples.

All analysis was done with R (V.4) using the pROC and Mosaic packages. STARD 2015 guidelines for diagnostic accuracy studies were followed.

This paper includes outcomes from all participants who had a baseline scan between study commencement (4 April 2019) and temporary closure for the Sars-CoV-2 pandemic on 18 March 2020 (n=11 566). Participants who had left the UK or who were withdrawn from the study and declined future review of cancer registry data were excluded from this analysis. Data were censored on 3 April 2023 (median follow-up from baseline LDCT 3.45 years, range 3.0–4.0).

### Role of the funding source

The funder was involved in study design but had no role in data analysis, data interpretation or writing of the report.

## Results

**Figure 1:**
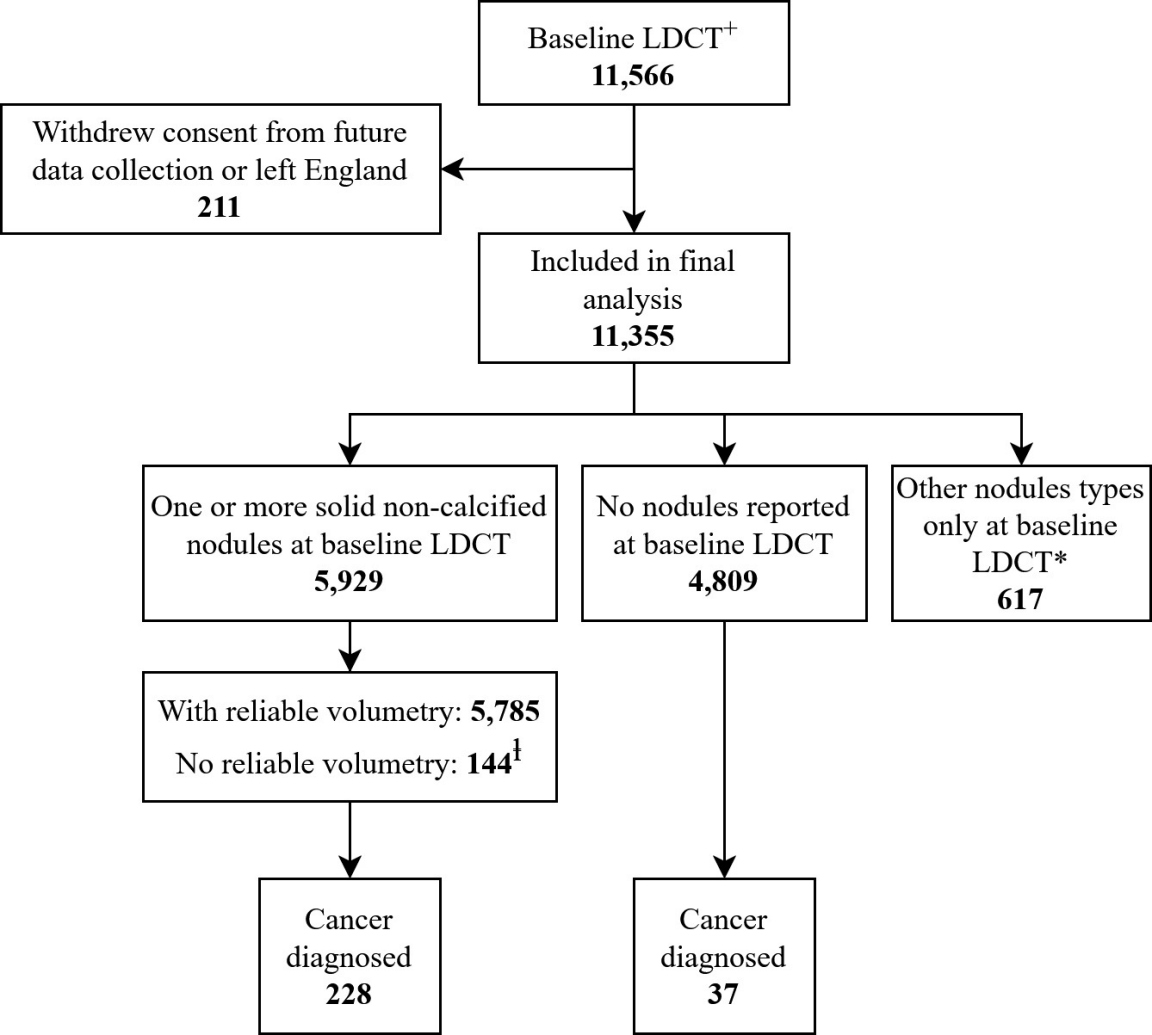
Data presented at a per-participant level. +Completed baseline LDCT in SUMMIT prior to April 2020. *Other nodule types reported in SUMMIT include subsolid, endobronchial and indeterminate focal consolidation. ^ⱡ^For the volumetric analysis, the 144 solid nodules with no reliable segmentation were included but were categorised by diameter thresholds of 6 mm for ‘rule out’ and 8 mm for ‘rule in’, reflecting how such nodules would be managed under BTS guidelines. LDCT, low-dose CT.

11 355 participants were included (figure 1). The median age (interquartile range) was 65.0 (60.0–70.0) years, 57.7% were male and median cumulative smoking history was 40.5 (32.3–51.0) pack years. The median follow-up period following baseline LDCT (via the SUMMIT Study and/or national cancer registries) was 3.45 (3.22–3.62) years, minimum follow-up period of 3.04 years.

Solid nodules reported at baseline LDCT in 228 of 5929 participants were subsequently proven malignant (crude risk 3.85%, 95% CI 3.37% to 4.37%). Lung cancer risk within the follow-up period in participants with no nodules reported at baseline CT was 0.77% (95% CI 0.54% to 1.06%).

In 144 participants (2.1%), reliable segmentation could not be achieved for the solid nodule, so they were analysed according to diameter (figure 1).

Malignancy risk of solid nodules increased with volume (crude OR 1.001/10 mm^3^ increase, p≤0.0001) and diameter (crude OR 1.225/1 mm increase, p≤0.0001).

### ‘Rule out’ thresholds

Solid nodules <6 mm diameter or <100 mm^3^ volume had equivalent risk of malignancy compared with participants with no nodules at baseline LDCT (risk of malignancy 0.88% and 0.81% vs 0.77%, p=0.7126 and p=0.9375, respectively). For the BTS ‘rule out’ thresholds of <80 mm^3^ or <5 mm, sensitivity was 86.8% (95% CI 81.8% to 90.9%) vs 93.4% (95% CI 89.4% to 96.3%, p=0.0003), specificity 65.4% (95% CI 64.1% to 66.7%) vs 24.6% (95% CI 23.5% to 25.8%, p<0.0001) and NPV 99.2% (95% CI 98.9% to 99.5%) vs 98.9% (95% CI 98.3% to 99.4%), respectively (table 1, online supplemental table s1). The <80 mm^3^ threshold encompassed 3756 participants (33.1% of the cohort) compared with 1412 (12.5%) of the cohort encompassed by the <5 mm threshold.

**Table 1:** Performance of rule-out diameter and volume thresholds for solid non-calcified nodules at baseline LDCT

Threshold	Cancers/participants	Risk (95% CI)	P value*	% of participants with any solid nodule at baseline LDCT included within threshold	Sensitivity (95% CI)	Specificity (95% CI)	Negative predictive value (95% CI)
No nodules	37/4809	0.77% (0.54 to 1.06)	–				
Any solid nodule	228/5929	3.85% (3.37 to 4.37)	<0.0001	100%	–	–	–
<5 mm	15/1421	1.06% (0.59 to 1.74)	0.3811	24.0%	93.4% (89.4 to 96.3)	24.6% (23.5 to 25.8)	98.9% (98.3 to 99.4)
<6 mm	21/2378	0.88% (0.55 to 1.35)	0.7126	40.1%	90.8% (86.3 to 94.2)	41.3% (40.1 to 42.6)	99.1% (98.7 to 99.5)
<80 mm^3^	30/3756	0.80% (0.54 to 1.14)	0.9766	63.3%	86.8% (81.8 to 90.9)	65.4% (64.1 to 66.7)	99.2% (98.9 to 99.5)
<100 mm^3^	34/4183	0.81% (0.56 to 1.13)	0.9375	70.6%	85.1% (79.8 to 89.4)	72.8% (71.6 to 73.9)	99.2% (98.9 to 99.4)

Data are at a per-participant level. Risk is calculated as total number of participants with lung cancer divided by the total number of participants with one or more solid nodules of that size or larger. Total baseline population undergoing baseline LDCT in this study was 11355.

* Value of p for 2 × 2 Pearson’s χ2 tests comparing crude malignancy risk for each diameter and volume threshold group to the no nodules group.

**Figure 2:**
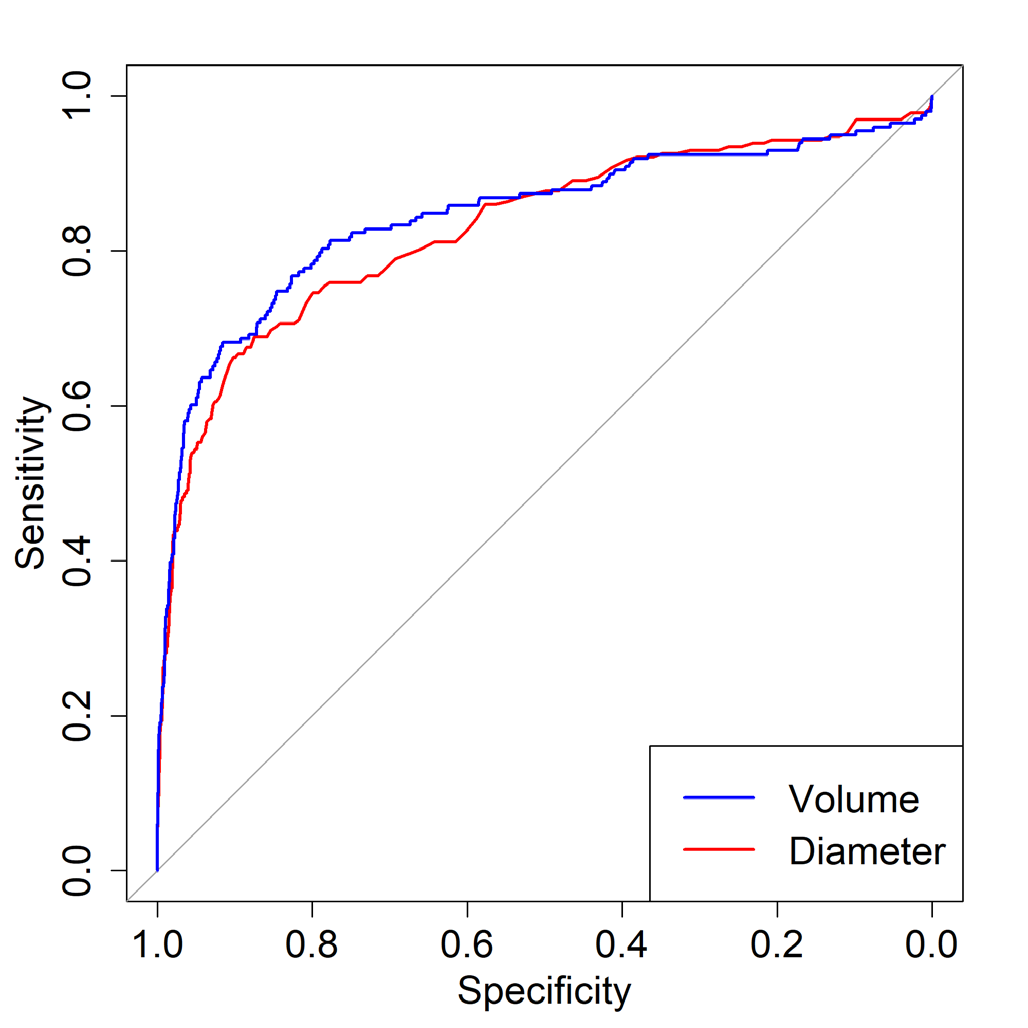
Receiver-operating characteristic curves for nodule diameter and volume. Area under the curve for diameter 0.822 (95% CI 0.787 to 0.857) and volume 0.844 (95% CI 0.807 to 0.8820), p=0.3852.

To compare sensitivity and specificity of volume and diameter assessments of nodule size at baseline scan in predicting malignancy, AUROCs were calculated. AUROC of diameter was 0.822 (95% CI 0.787 to 0.857), while AUC of volume (limited to those with reliable segmentation) was 0.844 (95% CI 0.807 to 0.882) (figure 2). There was no significant difference in AUROC (p=0.3852).

Compared with the 80 mm^3^ threshold used by BTS, the 100 mm^3^ threshold used by EUPS did not show a significant difference in net effect when analysed with our weighting criteria (net benefit +1.81; 95% CI −0.59 to +3.57), although including a further 427 participants (3.8% total population).

## ‘Rule In’ thresholds

Solid non-calcified nodules of ≥300 mm^3^ (or ≥8 mm diameter if reliable segmentation could not be achieved) with Brock Score ≥10% were referred for definitive assessment, while those with Brock Score <10% underwent interval CT. When size criteria alone were applied, a diameter threshold of ≥8 mm showed higher sensitivity than the volume threshold (77.6% (95% CI 71.7% to 82.9%) vs 66.7% (95% CI 60.1% to 72.8%, p<0.0001) respectively) at a cost of lower specificity 70.6% (95% CI 69.4% to 71.7%) vs 93.7% (95% CI 93.0% to 94.3%, p<0.0001) and PPV (9.5% (95% CI 8.2% to 11.0%) vs 29.7% (95% CI 25.8% to 33.9%)) (table 2, online supplemental tables s2.1 and s2.3). However, once the ≥10% additional Brock threshold was also considered, the differences between the volume and diameter thresholds in sensitivity, specificity or PPV were smaller (sensitivity 65.4% (95% CI 58.8% to 71.5%) vs 60.9% (95% CI 54.3% to 67.3%, p=0.0044), specificity 94.2% (95% CI 93.6% to 94.8%) vs 96.5% (95% CI 96.0% to 97.0%, p<0.0001), PPV 31.1% (95% CI 27.0% to 35.5%) vs 41.2% (95% CI 35.9% to 46.7%)) (table 2, online supplemental tables s2.2 and s2.4).

**Table 2:** Performance of ‘Rule in’ diameter and volume thresholds with and without Brock model threshold. Data are at a per-participant level. P value calculated by McNemar’s test

‘Rule in’ size threshold alone	≥8 mm	≥300 mm^3^	P value
Cancers/all participants with nodule meeting threshold	177/1855	152/511	
Sensitivity	77.6% (71.7–82.9)	66.7% (60.1–72.8)	<0.0001
Specificity	70.6% (69.4–71.7)	93.7% (93.0–94.3)	<0.0001
Positive predictive value	9.5% (8.2–11.0)	29.7% (25.8–33.9)	–

‘Rule in’ size threshold and Brock risk model ≥**10%**	≥8 mm and Brock ≥**10%**	≥300** mm^3^** and Brock ≥**10%**	
Cancers/all participants with nodule meeting threshold	149/479	139/337	
Sensitivity	65.4% (58.8–71.5)	60.9% (54.3–67.3)	0.0044
Specificity	94.2% (93.6–94.8)	96.5% (96.0–97.0)	<0.0001
Positive predictive value	31.1% (27.0–35.5)	41.2% (35.9–46.7)	–

We investigated the net effect of using a Brock risk prediction threshold for referral for PET-CT in addition to size threshold alone. Adding a 10% Brock threshold to the ≥8 mm diameter threshold gave a significant net benefit of +31.24 (95%CI 26.19 to 35.89). With a volume threshold of 300 mm^3^, adding a 10% Brock threshold gave no significant net benefit/disadvantage compared with size thresholds alone when analysed with our weighting criteria (net effect −0.29, 95%CI −3.46 to +2.56).

**Figure 3:**
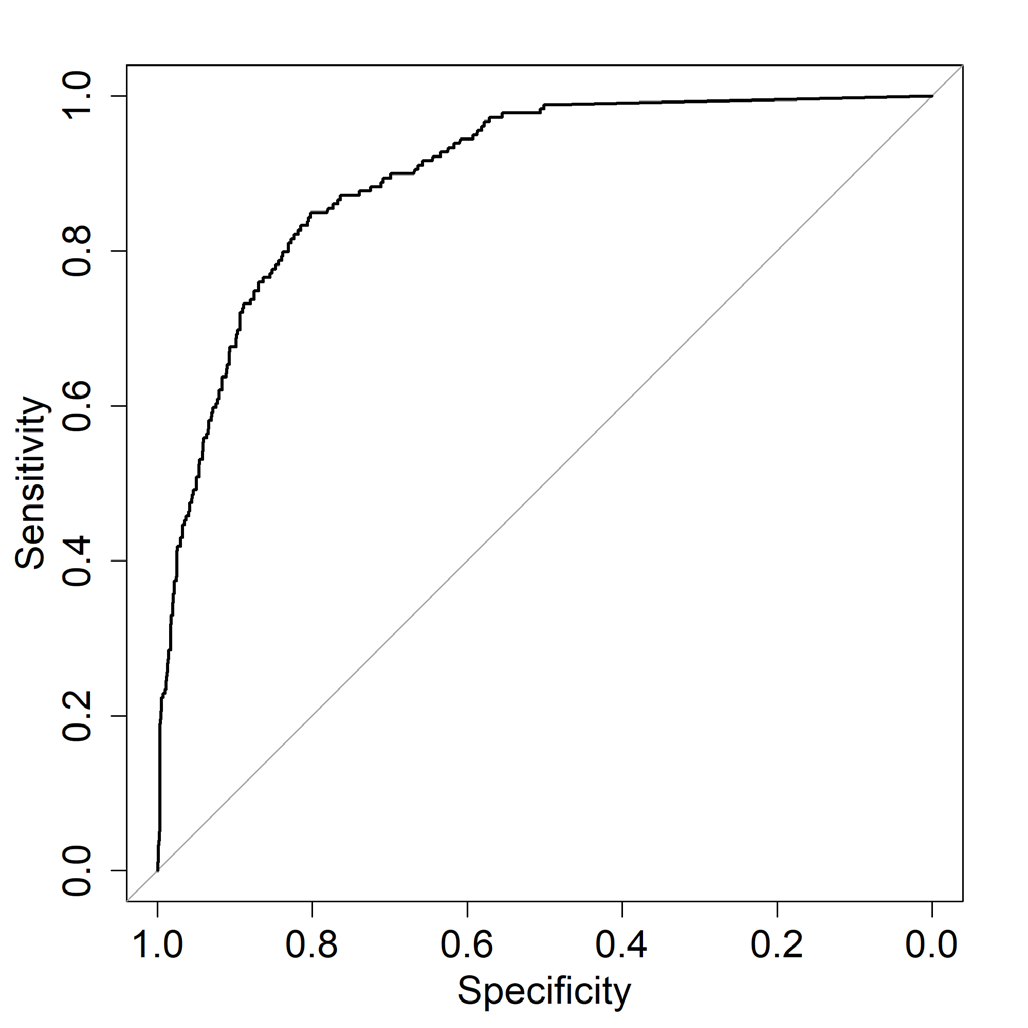
Receiver-operator characteristic curve for Brock model in predicting malignancy risk in solid nodules of >8 mm diameter. Area under the curve =0.898 (95% CI 0.8764 to 0.9196).

Finally, we explored whether changing the Brock threshold for referral from ≥10% to a mathematically determined ‘optimal’ threshold improved performance of the ‘rule in’ size threshold. Limiting analysis to solid nodules of ≥8 mm diameter (ie, the situation where the Brock Score is applied in BTS guidelines), an AUCROC for the Brock model of 0.898 (95% CI 0.876 to 0.920) was achieved (figure 3). Using Youden’s J statistic, an optimal Brock threshold of 9.905 was determined for predicting malignancy in solid nodules of ≥8 mm. Given the closeness to the currently used value of 10%, we did not perform a post hoc re-calculation of predicted performance at this threshold.

Although nodules with typical morphology of intrapulmonary lymph nodes were reported as a distinct nodule category (with no specific follow-up required^21^), such nodules were included in this analysis as non-calcified solid nodules. We identified four nodules labelled as perifissural at baseline LDCT which subsequently grew and were diagnosed as lung cancer within the study, and a further four participants with only perifissural nodules at baseline LDCT who were subsequently diagnosed with lung cancer outside of the study. Assuming the reported perifissural nodule was in fact malignant in all four of these cases, observing eight cancers in the 3823 participants with one or more perifissural nodules reported at baseline LDCT corresponds to a risk of 0.21% (95% CI 0.09% to 0.41%), lower than the risk of a subsequent lung cancer diagnosis in a participant with no nodules reported at baseline LDCT and hence supporting the guideline that no further follow-up is required for nodules of typical perifissural morphology.

## Discussion

Our results validate data from the Dutch–Belgian lung-cancer screening (NELSON) Trial of malignancy risk in nodules <100 mm^3^,^20^ supporting the finding that nodules below this size threshold confer no increased risk of malignancy compared with screening participants with no nodules reported at baseline LDCT. Our findings correspond closely to the risks given by Horeweg *et al*, although certain methodological differences (namely our analysis being limited to solid nodules only) should be acknowledged.^20^

We report the performance of the BTS 2015 nodule management guidelines when used in a screening context and compare the performance to the rule-out thresholds given by EUPS and single-dimension diameter thresholds. Significantly, our findings show that a volumetric threshold of 80 mm^3^ achieves a 41% higher specificity than a 5 mm diameter threshold, indicating a greatly reduced false positive rate. In practical terms, this means the 80 mm^3^ threshold encompasses a further 39% of participants with baseline solid nodules (equivalent to 20% of the total cohort) at baseline with an equal NPV; meaning an additional 20% of participants undergoing baseline LDCT can be discharged to the subsequent screening round. This increased specificity with similar sensitivity achieved with volume compared with diameter thresholds has been reported in previous studies into juxtapleural nodules.^22^ Our results show that while the vast majority of nodules can undergo accurate volumetric analysis, this is unavailable for a small proportion. This demonstrates a need for future nodule or TLHC protocols to continue to include diameter thresholds and underscores the need for radiologists reporting on such CADe-assisted programmes to assess the reliability of volumetric segmentation and decide if manual diameter measurements are required.

Furthermore, even with a conservative weighting of 50,^19^ we found no significant net disadvantage in raising the ‘rule out’ threshold from 80 mm^3^ (as per BTS) to 100 mm^3^, but an additional 4% of the baseline population included. At the level of population-level screening, such a change translates to a significant reduction in further scans (with the associated cost and participant anxiety). We chose this weighting on the assumption that these guidelines are applied in an annual/biennial screening programme and acknowledging the recognised costs (both financial and psychological^23^) of follow-up of indeterminate findings; however, the advantage of a net benefit analysis is that different weightings can be assigned using the same formula. For a screening programme, quantifying such net benefits pragmatically illustrates the magnitude of incremental benefit, that is, whether implementing new thresholds is worthwhile.

Conversely, the decision to refer from a screening programme for definitive investigation is associated with higher costs for both participant and health service. Optimising the PPV of cases referred for definitive assessment is therefore essential for efficient utilisation of hospital resources.^11^ Our net effect analysis found a risk score in addition to an 8 mm diameter threshold to determine referral for PET-CT (an aspect unique to BTS guidelines) was associated with a significant net benefit. This advantage wasn’t conferred when a 300 mm^3^ volume threshold was used as the higher specificity of the volumetric threshold meant a correspondingly smaller gain in specificity with the addition of a Brock Score. However, it is still worth noting that adding the Brock threshold reduced false positive MDT referrals by 45% (359 to 198, online supplemental table s2.3 and s2.4) at a cost of delayed diagnosis (at interval scan) for 8.6% (13 of 152) of cancers. Given the significant increase in both resource cost and anxiety associated with MDT discussion and PET-CT, such a trade-off may be justifiable, again assuming the safety net of a 3-month interval CT. Deriving an ‘optimal’ Brock threshold for solid nodules >8 mm diameter gave a cut-off of 9.905%, thus supporting the use of the 10% threshold in current BTS guidelines. When considering the sensitivity of the ≥8 mm/≥300 mm^3^ threshold of around 60%, it is important to remember that all nodules smaller than this (but larger than 5 mm/80 mm^3^) will undergo follow-up surveillance scan at 3 months, meaning true sensitivity of the protocol is that of the 5 mm/80 mm^3^ threshold (although with delays to some diagnoses). Data previously published by our group^24^ found that 88% of screen-detected lung cancers diagnosed following an interval scan performed at 3–6 months were diagnosed at stage 1, suggesting stage shift within this timeframe is relatively uncommon.

Research into this topic is limited by the fact that while prospective randomisation of participants into differing nodule management strategies is ideally warranted to truly compare performances, such studies would be challenging to perform (although one prospective study into nodule management is currently under way).^25^ As such, current data are primarily observational and come from large, standardised screening cohorts, with NELSON,^20^ the National Lung Screening Trial (NLST),^26^ Pan-Canadian^1^ and UK Lung Screen^4^ previously publishing nodule risk stratification models. Strengths of this paper include the size of our cohort, the standardised image acquisition, analysis and reporting, and that volume was measured by CADe analysis rather than derived from diameter. Furthermore, by restricting this analysis to solid nodules, this analysis is directly applicable to the scenarios where such thresholds will be applied (current guidelines advocate a different surveillance approach for subsolid nodules^7 27^).

There are limitations to our analysis. Nodules smaller than 4 mm diameter were below the quoted detection threshold for the CADe system and hence were not consistently reported (unless seen in retrospect when they subsequently grew and proved malignant); the risk of nodules <5 mm being malignant is therefore likely even lower than that given here. This analysis also required ascribing cancer outcomes to individual nodules, which was undertaken by comparing nodule location to biopsy and resection location. Therefore, while we are confident of a high degree of fidelity, we do not have the absolute certainty that fiducial placement prior to resection would have given. We acknowledge that the CIs in the sensitivity calculations are relatively wide; a consequence of the relatively small number of cancer diagnoses, even in a screening cohort of this size. Finally, our analysis used long-axis diameter (as advised by BTS) rather than average diameter advocated by Lung-RADS, meaning use of our results for direct comparison between the two approaches is not possible.

A limitation common to nodule risk prediction studies is that the binary outcome of malignancy fails to capture harm from false negative or false positive cases. In an annual or biennial screening programme, the safety net of further LDCTs reduces the risk that cancers presenting as sub-80 mm^3^ nodules at baseline LDCT progress to higher stage before diagnosis and must be balanced against the harms and costs of investigating benign disease^28^ ^29^ . Although determining the threshold for referral for definitive investigation depends on individual populations and healthcare systems, our data provide evidence to guide this discussion.

In conclusion, our findings provide evidence to support many aspects of current BTS guidelines. We show benefit from a volumetric ‘rule out’ threshold compared with diameter thresholds with increased specificity. We also show a clear benefit for the addition of a risk threshold to diameter alone for determining referral for MDT assessment, reducing false-positive referral rates within the safety net of a 3-month follow-up CT. Finally, we demonstrate that the current 10% is very close to the mathematically derived ‘optimal’ value for such a threshold. Our results also identify areas for potential changes. We validate findings from NELSON^20^ that nodules <100 mm^3^ were associated with no increased risk of malignancy and hence support a change to this threshold (already recommended by EUPS), with no net disadvantage but a further 4% of the baseline screening population included compared with the current 80 mm^3^ threshold. We do, however, acknowledge concerns about the variability between nodule segmentation software^30^ leading to a more conservative threshold.

Our results describe the performance of nodule management protocols applied to baseline LDCT in an asymptomatic high-risk cohort. Such evidence is essential in building a platform for the national targeted lung health check programme, as well as providing a benchmark against which novel imaging technologies can be compared.

## Supplementary material

10.1136/thorax-2024-222086 online supplemental file 1

10.1136/thorax-2024-222086 online supplemental file 2

## Data Availability

Data are available upon reasonable request.
