# Supplementary material for: Performance of volume and diameter thresholds in malignancy prediction of solid nodules in lung cancer screening
Source: Thorax. 2025 Jun 2;80(9):e222086. doi: 10.1136/thorax-2024-222086 (PMC12421124; doi:10.1136/thorax-2024-222086)
Supplement: online supplemental file 1 [file thorax-80-9-s001.docx]

**Appendix 1: 2x2 tables for performance metrics**

|  | **Diameter <5mm** | **Diameter ≥5mm** | **Total** |
| --- | --- | --- | --- |
| **Cancer** | 15 | 213 | 228 |
| **No cancer** | 1406 | 4295 | 5701 |
| **Total** | 1421 | 4508 | **5929** |

**S1.1: Diameter 5mm threshold – “Rule out”**

|  | **Diameter <6mm** | **Diameter ≥6mm** | **Total** |
| --- | --- | --- | --- |
| **Cancer** | 21 | 207 | 228 |
| **No cancer** | 2357 | 3344 | 5701 |
| **Total** | 2378 | 3551 | **5929** |

**S1.2: Diameter 6mm threshold – “Rule out”**

|  | **Diameter <80mm^3^** | **Diameter ≥80mm^3^** | **Total** |
| --- | --- | --- | --- |
| **Cancer** | 30 | 198 | 228 |
| **No cancer** | 3726 | 1975 | 5701 |
| **Total** | 3756 | 2173 | **5929** |

**S1.3 Volume 80mm^3^ threshold* – “Rule out”**

|  | **Diameter <100mm^3^** | **Diameter ≥100mm^3^** | **Total** |
| --- | --- | --- | --- |
| **Cancer** | 34 | 194 | 228 |
| **No cancer** | 4149 | 1552 | 5701 |
| **Total** | 4183 | 1746 | **5929** |

**Table s1.4: Volume 100mm^3^ threshold – “Rule out”**

|  | **Diameter <8mm** | **Diameter ≥8mm** | **Total** |
| --- | --- | --- | --- |
| **Cancer** | 51 | 177 | 228 |
| **No cancer** | 4023 | 1678 | 5701 |
| **Total** | 4074 | 1855 | **5929** |

**Table s2.1: Diameter ≥8mm “Rule in” threshold alone**

|  | **Diameter <8mm and/or Brock <10%** | **Diameter ≥8mm and Brock ≥10%** | **Total** |
| --- | --- | --- | --- |
| **Cancer** | 79 | 149 | 228 |
| **No cancer** | 5371 | 330 | 5701 |
| **Total** | 5450 | 479 | **5929** |

**Table s2.2: Diameter ≥8mm and Brock ≥10% “Rule in” thresholds**

|  | **Volume <300mm^3^** | **Volume ≥300mm^3^** | **Total** |
| --- | --- | --- | --- |
| **Cancer** | 76 | 152 | 228 |
| **No cancer** | 5342 | 359 | 5701 |
| **Total** | 541 | 511 | **5929** |

**Table s2.3 Volume >300mm “Rule in” alone**

|  | **Volume <300mm^3^ and/or Brock <10%** | **Volume ≥300mm^3^ and Brock ≥10%** | **Total** |
| --- | --- | --- | --- |
| **Cancer** | 89 | 139 | 228 |
| **No cancer** | 5503 | 198 | 5701 |
| **Total** | 5592 | 337 | **5929** |

**Table s2.4: Volume ≥300 and Brock ≥10% “Rule in”**
