# Supplementary material for: Performance of volume and diameter thresholds in malignancy prediction of solid nodules in lung cancer screening
Source: Thorax. 2025 Jun 2;80(9):e222086. doi: 10.1136/thorax-2024-222086 (PMC12421124; doi:10.1136/thorax-2024-222086)
Supplement: online supplemental file 2 [file thorax-80-9-s002.pptx]

## Slide 1
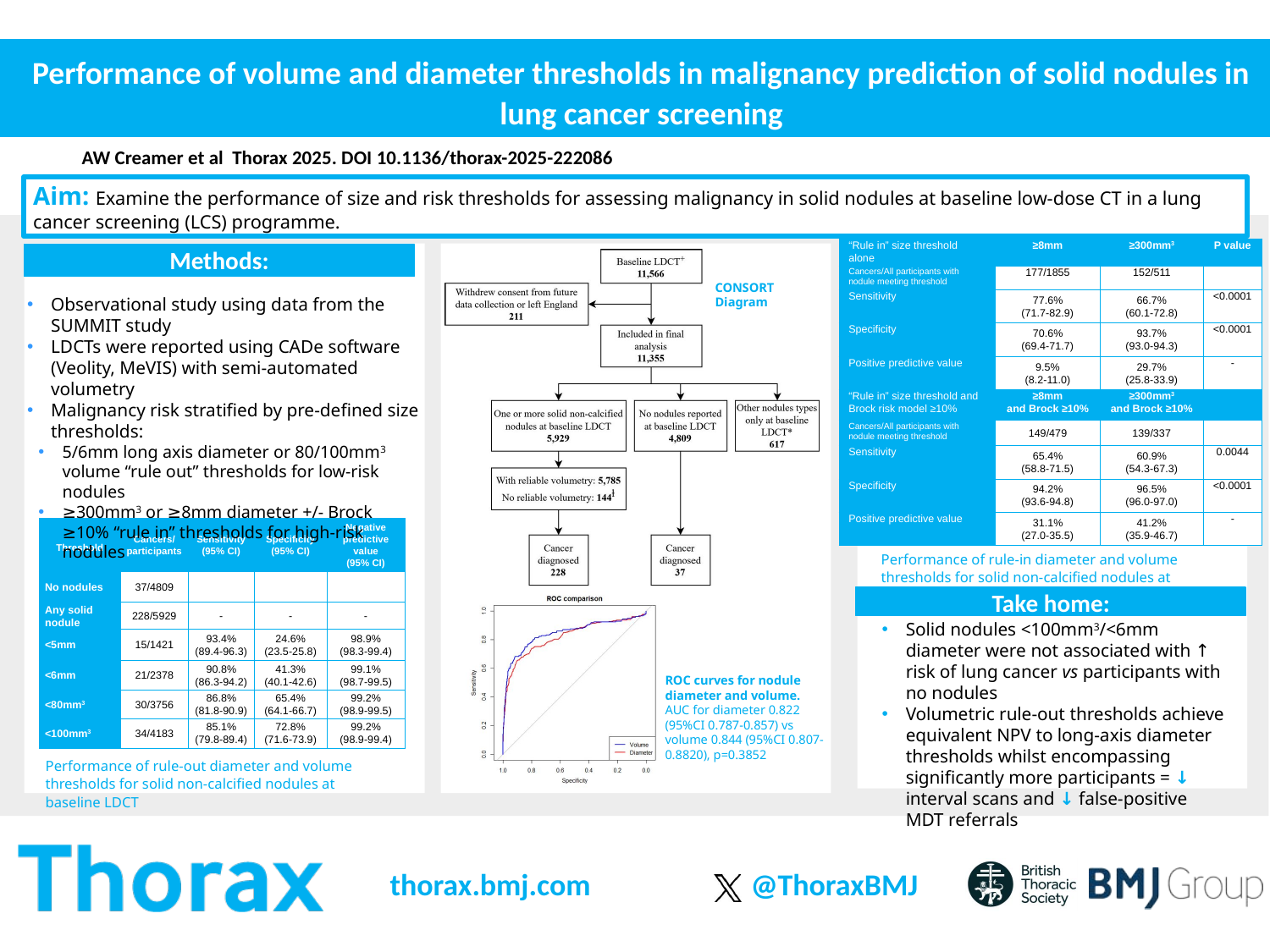

Performance of volume and diameter thresholds in malignancy prediction of solid nodules in lung cancer screening
AW Creamer et al Thorax 2025. DOI 10.1136/thorax-2025-222086
Aim: Examine the performance of size and risk thresholds for assessing malignancy in solid nodules at baseline low-dose CT in a lung cancer screening (LCS) programme.
Methods:
| “Rule in” size threshold alone | ≥8mm | ≥300mm3 | P value |
| --- | --- | --- | --- |
| Cancers/All participants with nodule meeting threshold | 177/1855 | 152/511 | |
| Sensitivity | 77.6% (71.7-82.9) | 66.7% (60.1-72.8) | <0.0001 |
| Specificity | 70.6% (69.4-71.7) | 93.7% (93.0-94.3) | <0.0001 |
| Positive predictive value | 9.5% (8.2-11.0) | 29.7% (25.8-33.9) | - |
| “Rule in” size threshold and Brock risk model ≥10% | ≥8mm and Brock ≥10% | ≥300mm3 and Brock ≥10% | |
| Cancers/All participants with nodule meeting threshold | 149/479 | 139/337 | |
| Sensitivity | 65.4% (58.8-71.5) | 60.9% (54.3-67.3) | 0.0044 |
| Specificity | 94.2% (93.6-94.8) | 96.5% (96.0-97.0) | <0.0001 |
| Positive predictive value | 31.1% (27.0-35.5) | 41.2% (35.9-46.7) | - |
CONSORT Diagram
Observational study using data from the SUMMIT study
LDCTs were reported using CADe software (Veolity, MeVIS) with semi-automated volumetry
Malignancy risk stratified by pre-defined size thresholds:
5/6mm long axis diameter or 80/100mm3 volume “rule out” thresholds for low-risk nodules
≥300mm3 or ≥8mm diameter +/- Brock ≥10% “rule in” thresholds for high-risk nodules
Manuscript Title
| Threshold | Cancers/ participants | Sensitivity (95% CI) | Specificity (95% CI) | Negative predictive value (95% CI) |
| --- | --- | --- | --- | --- |
| No nodules | 37/4809 | | | |
| Any solid nodule | 228/5929 | - | - | - |
| <5mm | 15/1421 | 93.4% (89.4-96.3) | 24.6% (23.5-25.8) | 98.9% (98.3-99.4) |
| <6mm | 21/2378 | 90.8% (86.3-94.2) | 41.3% (40.1-42.6) | 99.1% (98.7-99.5) |
| <80mm3 | 30/3756 | 86.8% (81.8-90.9) | 65.4% (64.1-66.7) | 99.2% (98.9-99.5) |
| <100mm3 | 34/4183 | 85.1% (79.8-89.4) | 72.8% (71.6-73.9) | 99.2% (98.9-99.4) |
Performance of rule-in diameter and volume thresholds for solid non-calcified nodules at baseline LDCT
Take home:
Solid nodules <100mm3/<6mm diameter were not associated with ↑ risk of lung cancer vs participants with no nodules
Volumetric rule-out thresholds achieve equivalent NPV to long-axis diameter thresholds whilst encompassing significantly more participants = ↓ interval scans and ↓ false-positive MDT referrals
ROC curves for nodule diameter and volume.
AUC for diameter 0.822 (95%CI 0.787-0.857) vs volume 0.844 (95%CI 0.807-0.8820), p=0.3852
© Author(s) (or their employer(s) 2019. Re-use permitted under CC BY. Published by BMJ.
Performance of rule-out diameter and volume thresholds for solid non-calcified nodules at baseline LDCT
thorax.bmj.com @ThoraxBMJ
